# Age-Dependent Oxidative DNA Damage Does Not Correlate with Reduced Proliferation of Cardiomyocytes in Humans

**DOI:** 10.1371/journal.pone.0170351

**Published:** 2017-01-18

**Authors:** Yanhui Huang, Haifa Hong, Minghui Li, Jinfen Liu, Chuan Jiang, Haibo Zhang, Lincai Ye, Jinghao Zheng

**Affiliations:** 1 Department of anesthesiology, Shanghai Children’s Medical Center, Shanghai Jiaotong University School of Medicine, Shanghai, China; 2 Department of Thoracic and Cardiovascular Surgery, Shanghai Children’s Medical Center, Shanghai Jiaotong University School of Medicine, Shanghai, China; 3 Shanghai Institute of Pediatric Congenital Heart Diseases, Shanghai Children’s Medical Center, Shanghai Jiaotong University School of Medicine, Shanghai, China; 4 Institute of Pediatric Translational Medicine, Shanghai Children’s Medical Center, Shanghai Jiaotong University School of Medicine, Shanghai, China; Cincinnati Children's Hospital Medical Center, UNITED STATES

## Abstract

**Background:**

Postnatal human cardiomyocyte proliferation declines rapidly with age, which has been suggested to be correlated with increases in oxidative DNA damage in mice and plays an important role in regulating cardiomyocyte proliferation. However, the relationship between oxidative DNA damage and age in humans is unclear.

**Methods:**

Sixty right ventricular outflow myocardial tissue specimens were obtained from ventricular septal defect infant patients during routine congenital cardiac surgery. These specimens were divided into three groups based on age: group A (age 0–6 months), group B (age, 7–12 months), and group C (>12 months). Each tissue specimen was subjected to DNA extraction, RNA extraction, and immunofluorescence.

**Results:**

Immunofluorescence and qRT-PCR analysis revealed that DNA damage markers—mitochondrial DNA copy number, oxoguanine 8, and phosphorylated ataxia telangiectasia mutated—were highest in Group B. However immunofluorescence and qRT-PCR demonstrated that two cell proliferation markers, Ki67 and cyclin D2, were decreased with age. In addition, wheat germ agglutinin-staining indicated that the average size of cardiomyocytes increased with age.

**Conclusions:**

Oxidative DNA damage of cardiomyocytes was not correlated positively with age in human beings. Oxidative DNA damage is unable to fully explain the reduced proliferation of human cardiomyocytes.

## Introduction

Cardiomyocyte deficiency underlies most causes of heart failure, which is the most common cause of disease-related deaths among citizens within industrialized nations [1]. Cardiomyocyte deficiency is mostly due to limited amounts of proliferating cardiomyocytes, which are not sufficient for ameliorating contractile dysfunction [2]. Thus, there is a profound demand to replenish the cardiomyocytes that are lost during heart failure by either promoting the proliferation of endogenous cardiomyocytes or facilitating stem cell differentiation (3,4)[3,4]. The percentage of proliferating cardiomyocytes in mammals declines with age, especially past the neonatal period, during which both humans and rodents have more proliferating cardiomyocytes than in any other period [5, 6]. The mechanism that underlies this reduced proliferation of cardiomyocytes remains unknown.

Reduced levels of proliferating cardiomyocytes could be due to oxidative DNA damage. Puente et al. observed that the oxygen-rich postnatal environment induces mouse cardiomyocyte cell-cycle arrest through DNA-damage [7] and that cycling cardiomyocytes in adults exist within a relatively hypoxic environment [8]. Further, ventricular assist devices are able to aid in heart function recovery by reducing oxidative damage to DNA [9]. These findings suggest that oxidative DNA damage might be responsible for the correlation between decreased cardiomyocytes proliferation of and age. In addition, age was also associated with an increase in mouse heart DNA oxidative damage markers: mitochondria DNA (mtDNA), oxoguanine 8 (8-oxoG), and phosphorylated ataxia telangiectasia mutated (p-ATM) [7]. However, there is little information available regarding similar amounts of oxidative DNA damage in human heart.

In humans, the mitochondrion is considered to be the major production site of free radicals, which can lead to DNA damage [10]. The presence of mtDNA is considered to be an indicator of mitochondrion damage[7,9], while 8-oxoG is an extremely common DNA lesion that caused by reactive oxygen species [11] and can promote mismatched pairings with adenine, resulting in G to T and C to A substitutions within the genome [12]. Ataxia telangiectasia mutated (ATM), a serine/threonine protein kinase that is activated by DNA double strand breaks, phosphorylates several key proteins activate DNA damage checkpoints, and subsequently leads to cell cycle arrest or DNA repair. [13], suggesting that p-ATM is an essential mediator in activating the DNA damage response [7]. In this study, mtDNA copy number, 8-oxoG, and p-ATM were quantified from human hearts and assessed for correlation with age. Proliferating markers Ki67 and cyclin D2 were also detected in order to determine a correlation between cardiomyocyte proliferation and oxidative DNA damage.

## Materials and Methods

### Study population and tissue sampling

All patients who were diagnosed as having a ventricular septal defect (VSD) with right ventricular outflow tract hypertrophy were selected for investigation at the Shanghai Children’s Medical Center between January 2016 and September 2016. A total of sixty-four patients were included in the study—sixty were male and four were female. In order to balance the study, the four female samples were excluded and sixty male right ventricular outflow myocardial tissue specimens were collected during required resections in relieving obstructions from infant VSD (S1 Table). Each specimen was preserved in liquid nitrogen and later divided into three portions, which were used for DNA extraction, quantitative real-time polymerase chain reaction (qRT-PCR), and immunofluorescence. The Animal Welfare and Human Studies Committee at the Shanghai Jiaotong University School of Medicine approved all procedures and parental written informed consent was obtained prior to initiating the study.

### mtDNA quantification by real-time polymerase chain reaction (RT-PCR)

For mtDNA quantification [9], DNA was extracted and purified from tissue samples directly following proteinase K digestion and phenol:chloroform extraction. mtDNA was quantified using RT-PCR with primers that targeted a relatively stable site in the mitochondrial DNA minimal arc [10]: mtDNA F- CTAAATAGCCCACACGTTCCC; and mtDNA R- AGAGCTCCCGTGAGTGGTTA. Total nuclear DNA was also quantified using RT-PCR with the following primers that targeted single-copy nuclear DNA within the beta-2M gene [9]: nuclear DNA F- GCTGGGTAGCTCTAAACAATGTATTCA and nuclear DNA R- CCATGT ACTAACAAATGTCTAAAATGGT. Quantification was performed using a SYBR Green PCR Master Mix and a 7900 Sequence Detection System (Applied Biosystems, Foster City, California). The relative mtDNA copy number was calculated from the ratio of mtDNA copies to nuclear DNA copies per gram of tissue. The relative fold change was then calculated using the ΔΔCT method.

### Immunostaining and quantification of oxidative DNA damage marker 8-oxoG and p-ATM

VSD heart tissue(0.3 cm × 0.2 cm × 0.1 cm) were fixed in 4% paraformaldehyde in phosphate buffer solution (PBS) overnight at 4°C, dehydrated with 30% sucrose in PBS overnight at 4°C, and frozen in Tissue-Tek optimum cutting temperature compound on a liquid nitrogen-cooled metal block before being cut into 8 μm cryosections. The sections were mounted on coverslips and used for 8-oxoG and p-ATM staining. The initial slide was selected using a random number generator and every sixth slide after was chosen for microscopy by systematic random selection. The slides were washed three times with phosphate buffer solution (PBS) and then permeabilized for 15 min with 5% Triton X-100. After washing with PBS three times, the slides were incubated overnight at 4°C with a rabbit antibody against cardiac sarcomeric alpha actinin (Abcam, ab90776, 1:200) and a mouse monoclonal anti-8-oxoG antibody (Abcam, ab64548, 1:200) or a mouse antibody against cardiac troponin T (Abcam, ab8295, 1:100) and a rabbit antibody against p-ATM (Abcam, ab81292, 1:100). The slides were then incubated for 30 min with an Alexa Fluor® 555-conjugated anti-rabbit secondary antibody (CST, 4409, 1:500) and an Alexa Fluor® 488-conjugated anti-mouse secondary antibody (Abcam, ab150073, 1:500). Nuclei were stained with 4',6-diamidino-2-phenylindole(DAPI,R37606, life technologies). For quantification, 8-oxoG and pATM foci were counted using the spot detection function of the Imaris software (Bitplane, South Windsor, Connecticut) [9]. For each sample, the average number of foci per myocyte was quantified using the images from 10 fields.

### Laser scanning confocal microscopy analysis of Ki67 and cyclin D2 expression

VSD heart tissue sections (8μm) were used for Ki67 and cyclin D2 staining. The initial slide was selected using a random number generator and every seventh slide after was chosen for microscopy by systematic random selection. The slides were stained overnight with mouse monoclonal antibodies against cardiac troponin T (Abcam, ab8295, 1:200) and an Alexa Fluor® 488-conjugated rabbit anti-Ki67 antibodies (Abcam, ab154201; 1:200) or rabbit antibody against cardiac sarcomeric alpha actinin (Abcam, ab90776, 1:200) and mouse antibody against cyclin D2(Abcam, ab3085,1:200). After three washes, the sections were incubated with Alexa Fluor® 555-conjugated anti-mouse secondary antibodies (Abcam, ab150107; 1:200) or Alexa Fluor® 555-conjugated anti-rabbit secondary antibody (CST, 4409, 1:500) and an Alexa Fluor® 488-conjugated anti-mouse secondary antibody (Abcam, ab150073, 1:500) for 30 min. Three researchers who were blinded to sample identities performed the quantification of cellular Ki67 and cyclin D2 events using either manual counting or digital thresholding (image segmentation and the creation of a binary image from a gray scale) methods. Analysis of the converted binary images was performed using ImageJ (National Institutes of Health).

### Ki67 and cyclin D2 quantification by qRT-PCR analysis

Total RNA was extracted using Trizol (Invitrogen) reagent. RT-PCR was performed using a PrimeScriptTM reagent kit (Takara, Japan). qRT-PCR was performed using a SYBR Green Power Premix Kit (ABI) according to the manufacturer’s instructions. The qRT-PCR reactions were performed in an Applied Biosystems 7900 Fast Real-Time PCR System and the following conditions were utilized: 1 cycle at 95°C for 10 s, followed by 40 cycles of 95°C for 15 s and 60°C for 60 s. The primers were obtained from Generay Bio (China). The sequences were as follows: Ki67 forward primer- 5’-ACTTCCCCATGTCTCCAAGG-3’; Ki67 reverse primer- 5’-GCAGTGGTATCAACGCAGAG-3’; GAPDH forward primer- 5’-TGCACCACCAACTGCTTAGC-3’; GAPDH reverse primer- 5’-GGCATGGACTGTGGTCATGAG-3’; Cyclin D2 forward primer- 5’-GCAAATGTGTACGTGCATGC-3’; and Cyclin D2 reverse primer- 5’-CGATGATTTGCTGGGGATG-3’.

### Cell size measurement by wheat germ agglutinin staining

Following antigen retrieval, slides were rinsed three times in PBS and then incubated for 10 min at room temperature with wheat germ agglutinin conjugated to Alexa Fluor 488 (MP00831, Life Technologies), as described by the manufacturer. The slides were subsequently washed three times with PBS and incubated overnight at 4°C with an anti-troponin T antibody (Abcam, ab8295, 1:200). The following day, the slides were rinsed three times with PBS and then incubated for 30 min at room temperature with Alexa Fluor® 555-conjugated anti-mouse secondary antibodies (Abcam, ab150107; 1:200). After washing three times with PBS, the slides were mounted in Vectashield (Vector Laboratories, Burlingame, California). In order to quantify cell size, images were captured and ImageJ was used to determine the area of each cell. Quantitative analysis involved counting multiple fields from six independent samples for each group (100 cells per field were assessed from a total of 1000 cells per group).

### Statistical analysis

Continuous data—including age, weight, protein expression, mRNA levels, and the number of Ki67-positive cells—were expressed as mean ± standard deviation. One-way ANOVA and SNK were used to determine statistical significance. Categorical variables were expressed using counts and percentages, which were compared for survival and death using Fisher’s exact test. *P*-values < 0.05 were considered to be statistically significant. Statistical analyses were performed using SAS software version 9.2 (SAS Institute Inc., Cary, NC, USA).

## Results

### Baseline patient characteristics

Sixty infants diagnosed with VSD (S1 Fig) that had right ventricular outflow tract muscle bundle thickening were included in this study. As oxygen saturation and pressure load have an impact on cardiomyocyte proliferation [7–9], patients were selected to ensure that there were no differences in oxygen saturation, gender, or pulmonary arterial pressure (increasing right ventricular pressure load) among the three groups (Table 1). As neuregulin1 has been demonstrated to stimulate the proliferation of human cardiomyocytes that are < 6 mo old [14], the sixty heart samples were divided into three groups based on age: Group A, 0–6 mo; Group B, 7–12 mo; and Group C, >12 mo. The twenty patients from each group were analyzed to determine putative correlations between age and oxidative DNA damage in human hearts. The patient characteristics were deemed to be comparable and suitable for studying the effects of age on cardiomyocyte oxidative DNA damage.

**Table 1:** Patients’ clinical information (Individual information provided in S1 Table).

	Group A	Group B	Group C	*P*
Age(mo)	3.5 ± 0.4	8.3 ± 0.9	18.5 ± 3.8	<0.001**
Body weight(kg)	5.2±0.9	6.7±0.3	12.2±0.5	<0.001**
Oxygen saturation	99.5±0.6%	99.5±0.7%	99.7±0.6%	1
Pulmonary arterial pressure	33.4 ± 5.7	33.2 ± 4.6	29.8 ± 4.6	0.4673

Mean ± standard deviation

**p < 0.01, ANOVA, SNK, n = 20.

### Oxidative DNA damage in hearts from different ages analyzed by mtDNA content

**Fig 1:**
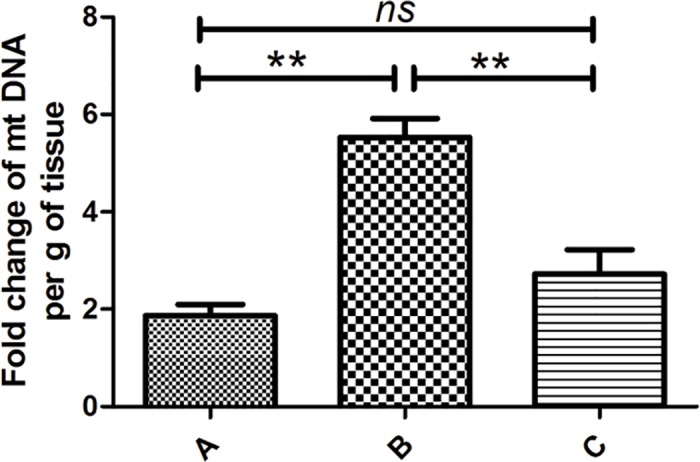
Oxidative DNA damage in different age hearts analyzed by mtDNA copy number. **q**RT-PCR was used to analyze the levels of mtDNA in VSD patient hearts. mtDNA was not positively correlated with age, although group B (7–12 mo) had the highest amount of mtDNA. Bars indicate mean ±standard deviation. Both ANOVA and SNK were performed to evaluate statistical significance, n = 20, **p < 0.01.

The ratio of mtDNA to nuclear DNA is often used to estimate the mtDNA copy number [14] and this serves as a high-level indicator of mitochondrial biogenesis [15], which is also considered to be a marker for oxidative DNA damage [7,9]. mtDNA copy number was measured in hearts from the three groups and the average fold change of mtDNA in groups A, B, and C were 2.72 ± 1.12, 5.52 ± 0.78, and 1.87 ± 0.74, respectively (p < 0.01, n = 20, Fig 1), suggesting that mtDNA content in human heart did not increase with age, although Group B (7–12 mo) did have the highest mtDNA copy number. These results are distinct from those obtained in mice, in which the mtDNA content of heart increased with age [7].

### Oxidative DNA damage in hearts from different ages analyzed by 8-oxoG levels

**Fig 2:**
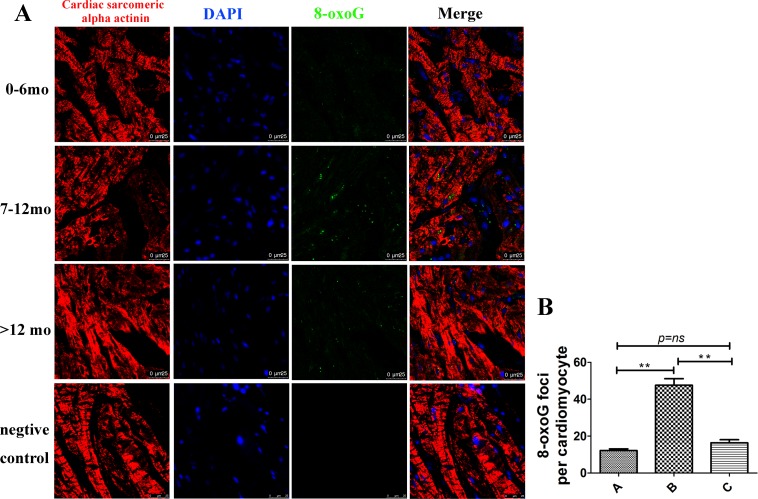
Oxidative DNA damage in hearts from different ages analyzed by 8-oxoG. (A) Representative graph of groups A, B, and C. 8-oxoG was not positively correlated with age, and group B (7–12 mo) had the highest amount of 8-oxoG. Cardiac sarcomeric alpha actinin (red), 8-oxoG (green), and DAPI (blue) staining; Scale bar = 25 μm. (B) Quantification of 8-oxoG foci per cardiomyocyte in each group, n = 20, **p < 0.01.

Increased levels of mtDNA are often correlated with increased amounts of oxidative DNA damage. In order to validate the results observed with mtDNA copy number, 8-oxoG levels were measured from the three groups [12]. 8-oxoG levels were significantly increased in Group B, and 8-oxoG signaling was observed in or near the cell nucleus in both cardiomyocytes and non-cardiomyocytes (Fig 2). These results indicated that oxidative DNA damage was highest in group B (7–12 mo).

### Oxidative DNA damage in hearts from different ages analyzed by p-ATM

**Fig 3:**
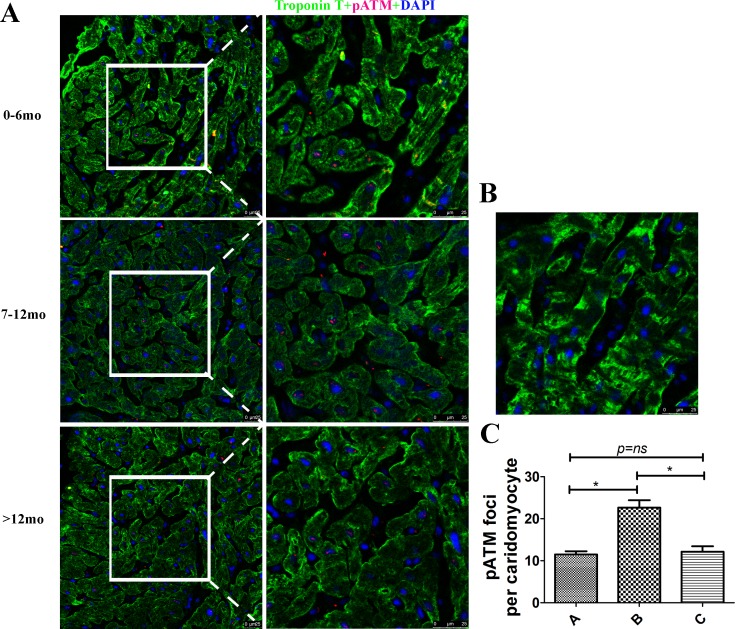
Oxidative DNA damage in hearts from different ages analyzed by p-ATM. (A) Representative graph of groups A, B, and C. p-ATM was not positively correlated with ages, and group B (7–12 mo) had the highest amount of p-ATM. Cardiac troponin T (green), p-ATM (red), and DAPI (blue) staining; Scale bar = 25 μm. (B) Negtive control. Primary antibody was substituted with PBS. (C) Quantification of p-ATM foci per cardiomyocyte in each group, n = 20, **p < 0.01.

ATM is the primary DNA double-strand break-sensor protein and autophosphorylation at residue Ser1981 plays a central role in cell cycle delay after DNA damage [13]. In order to further confirm that Group B had higher levels of DNA damage, p-ATM levels were measured in hearts from different ages. p-ATM was expressed only in the nucleus and was significantly increased in Group B (Fig 3), further confirming that oxidative DNA damage was highest in group B (7–12 mo).

### Proliferation activity of cardiomyocytes from different age groups

**Fig 4:**
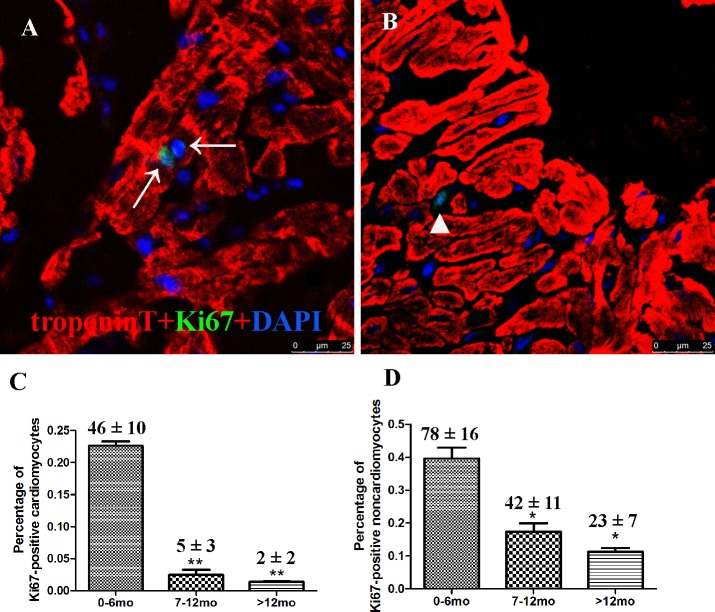
Cardiomyocyte proliferation from different age groups analyzed via confocal microscopy. Confocal microscopy of tissue sections using Ki67 and troponin T indicated that proliferating cardiomyocytes were decreased relative to age. (A) Representative Ki67-positive cardiomyocytes in Group B. (B) Representative Ki67-positive non-cardiomyocytes in Group B. (C) Quantification of Ki67-positive cardiomyocytes. (D) Quantification of Ki67-positive non-cardiomyocytes. Data presented as mean ±standard devation; *p < 0.05, **p < 0.01. Cardiac troponin T (red), Ki67 (green), and DAPI (blue) staining are shown. Arrows indicate proliferating cardiomyocytes and the triangle indicates non-cardiomyocytes.

Ki67 is present during all active phases of the cell cycle (G1, S, G2 and mitosis) and serves as an excellent marker for cell proliferation. Thus, Ki67-positive cells were measured from all three groups, resulting in 46 ± 10, 5 ± 3, and 2 ± 2 Ki67-positive cardiomyocytes (troponin T positive) in groups A, B, and C, respectively, (*p* = 0.021, n = 20). The number of Ki67-positive non-cardiomyocytes (troponin T negative) in the three groups was 78 ± 16, 42 ± 11, and 23 ± 7, respectively (p = 0.0015, n = 20). A total of 20,124 ± 1442, 20,344 ± 3463, and 21123 ± 1463 cells were counted for groups A, B, and C (n = 20) and the percentage of Ki67-positive cardiomyocytes in each group was 0.23 ± 0.05, 0.02 ± 0.01, and 0.01±0.01, respectively (*p* < 0.05). The percentage of Ki67-positive non-cardiomyocytes in each group was 0.39 ± 0.06, 0.17 ± 0.04, and 0.11 ± 0.02, respectively (*p* < 0.05; Fig 4). These results indicated that both cardiomyocyte and non-cardiomyocyte proliferation activities were decreased with age, which is consistent with previous reports [6,16].

**Fig 5:**
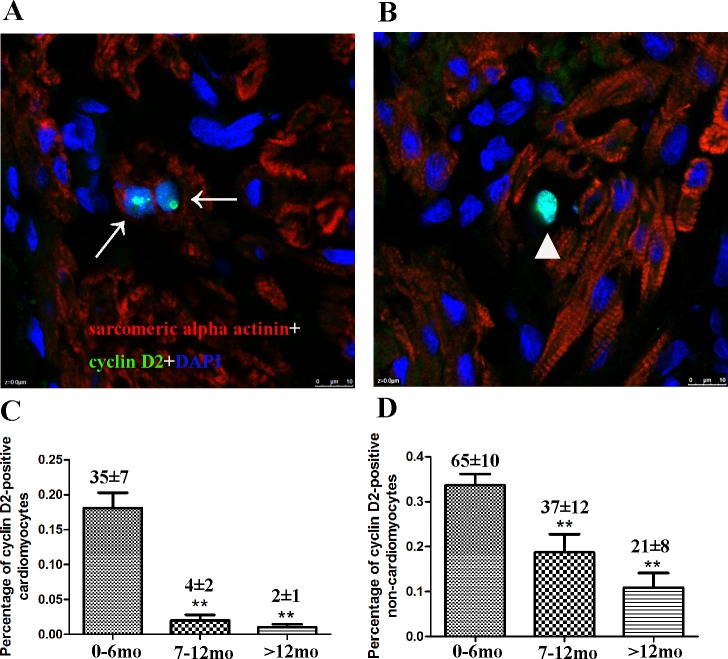
Cardiomyocyte proliferation from different age groups analyzed by cyclin D2. Confocal microscopy of tissue sections using cyclin D2 and cardiac sarcomeric alpha actinin indicated that proliferating cardiomyocytes were decreased relative to age. (A) Representative cyclin D2-positive cardiomyocytes in Group B. (B) Representative cyclin D2-positive non-cardiomyocytes in Group B. (C) Quantification of cyclin D2-positive cardiomyocytes. (D) Quantification of cyclin D2-positive non-cardiomyocytes. Data presented as mean ± standard deviation; *p < 0.05, **p < 0.01. cardiac sarcomeric alpha actinin (red), cyclin D2 (green), and DAPI (blue) staining are shown. Arrows indicate proliferating cardiomyocytes and triangle indicates non-cardiomyocytes.

Immunofluorescence was performed to detect and quantify cyclin D2-positive cardiomyocytes. The number of cyclin D2-positive cardiomyocytes (sarcomeric alpha actinin positive) in the three groups was 35 ± 7, 4 ± 2, and 2± 1, respectively (*p* = 0.003, n = 20) and the number of cyclin D2-positive non-cardiomyocytes (sarcomeric alpha actinin negative) in the three groups was 65 ± 10, 37 ± 12, and 21 ± 8, respectively (*p* = 0.005, n = 20), A total of 19,223 ± 1553, 19445 ± 2212, and 19023 ± 1654 cells were counted for groups A, B, and C (n = 20) and the percentage of Ki67-positive cardiomyocytes in each group was 0.18 ± 0.02, 0.02 ± 0.01, and 0.01 ± 0.01, respectively (*p* < 0.01). The percentage of Ki67-positive non-cardiomyocytes in each group was 0.34 ± 0.02, 0.02 ± 0.01, and 0.01 ± 0.01, respectively (*p* < 0.01; Fig 5).

**Fig 6:**
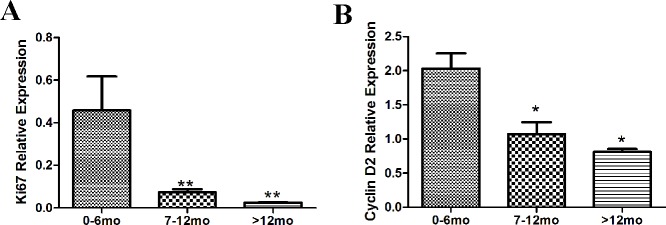
Ki67 and cyclin D2 mRNA is significantly decreased relative to age. qRT-PCR was used to analyze Ki67 and cyclin D2 mRNA levels. Both Ki67 (A) and cyclin D2 (B) were decreased with age. GAPDH served as a control. Bars indicate mean ±standard deviation. * *p* < 0.05, **p < 0.01, n = 20.

In order to further support the above results, qRT-PCR was performed to detect the mRNA levels of Ki67 and cyclin D2. The relative expression of Ki67 and cyclin D2 decreased with age (Fig 6), confirming that cardiac cell proliferation activity decreased with age.

### Cell size determined by WGA

**Fig 7:**
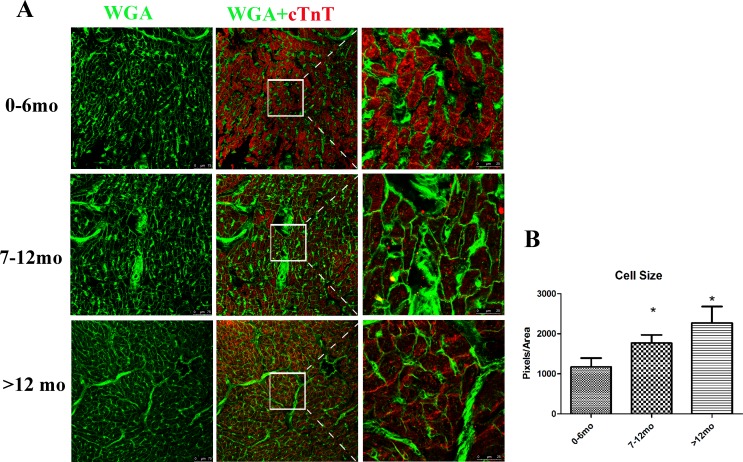
Cell size increased with age. (A) Representative graph of each group. Cardiac troponin T (red), WGA (green). (B) Quantification of the cell size for each group. Data presented as mean ±standard deviation; ***p < 0.001, n = 1000 cells for each group.

Previously, hypertrophy was noted to act as an alternative method by which postnatal heart growth and pathological hypertrophy of cardiomyocytes affected heart function [17]. Thus, the relationship between cardiomyocyte hypertrophy and age was assessed. WGA selectively binds to both N-acetylglucosamine and N-acetylneuraminic acid (sialic acid) residues, and serves as an effective probe for visualizing cell boundaries. An Alexa Fluor 488 conjugate of WGA was used to visualize cardiomyocyte boundaries, and the size of cardiomyocytes was found to increase with age (Fig 7), consistent with previous reports [1]. This suggested that hypertrophy was an alternative growth method when cardiomyocytes exit proliferation [18].

## Discussion

Cardiomyocyte proliferation contributes to postnatal heart growth in young humans and is very important in recovering heart function [16]. However mammalian cardiomyocytes lose proliferation activities during the aging process and cycling cardiomyocytes are almost undetectable by adulthood [16]. These limited numbers of cycling cardiomyocytes renders heart regeneration and functional recovery to be nearly impossible in adults, especially as the underling mechanism for the loss of mammalian cardiomyocyte proliferation remains to be elucidated. Currently, there are two possible explanations for this phenomenon: 1) The well-organized contractile architecture of adult cardiomyocytes may physically encumber cell division [2] and 2) Cardiomyocyte centrosomal integrity is lost shortly after birth, which contributes to a post-mitotic state of mammalian cardiomyocytes [19], as DNA integrity is a critical factor that impacts cell cycle activity [7,9]. However, these studies were performed using animal models and there are currently no data obtained from human studies. This study is the first report to investigate the relationship between DNA integrity and cardiomyocyte proliferation in humans.

All samples were obtained from the thickening muscle bundle of VSD patients. Although previous studies have indicated that there is no significant difference in the number of Ki67-positive cardiomyocytes between normal and VSD hearts [20], the effect of VSD or muscle thickening on oxidative damage remains unclear. Therefore, caution should be applied in directly translating these results to healthy human hearts. However understanding the relationship between DNA integrity and cardiomyocyte proliferation in VSD hearts is beneficial in understanding the regulatory network of heart regeneration.

This study also examined oxidative DNA damage in human heart, which did not linearly increase with age but instead reached a peak before decreasing to original levels. This result was consistent with Murray et al. [21], but contrary to Puente et al. [7], which could be explained in two possible ways. First, oxidative DNA damage does not reduce cardiomyocyte proliferation during postnatal heart development, while increased oxygen free radical production may promote cardiomyocyte proliferation via ERK1/2 and c-Myc-dependent activation of cyclin D2 transcription [21–22]. Interestingly, both Mollova et al. and Bergmann et al. demonstrated that human cardiomyocyte proliferation continues throughout the first 10 years of human development [23–24]. Oxidative DNA damage alone cannot explain this phenomenon. The second explanation may be that oxidative DNA damage in human heart is different from mice. For example, there are junction molecules, such as TWINKLE helicase, that emerge in human heart mtDNA during postnatal development and these molecules are absent in mouse hearts, concomitant with an increased demand for oxidative metabolism [25]. Pohjoismäki et al. demonstrated that overexpression of TWINKLE helicase in mice was able to protect cardiomyocytes from genotoxic stress caused by reactive oxygen species [25]. These results indicate that TWINKLE maintains mtDNA integrity to promote cardiomyocyte survival, suggesting that the human heart is more tolerant to reactive oxygen species than mouse heart. The data from this study demonstrated that oxidative DNA damage in human cardiomyocytes reaches a peak at 7–12 mo, followed by a surprising decrease to original levels, indicating that DNA damage repair in human cardiomyocytes is more efficacious than in rodents.

Although human cardiomyocytes are more tolerant to reactive oxygen species and may have greater oxidative repair capacity [25], the loss of cell cycling activity is similar to mouse cardiomyocytes [6, 16], which could be due to two possible explanations: 1) Oxidative DNA damage after birth causes permanent cell cycle arrest and subsequent recovery cannot reverse this process, and 2) Oxidative DNA damage only contributes to one component of cell cycle arrest. At birth, under increased hemodynamic stress, neonatal cardiomyocytes undergo cytoskeletal architecture reorganization in order to adjust to postnatal physical stresses. Subsequent cytoskeletal reorganization of cardiomyocytes could impact centrosomal integrity and hamper proliferation [19]. Supporting these findings, mouse models have been used to demonstrate that proliferating cardiomyocytes are the same as striated muscle-disassembled cardiomyocytes [2]. Thus, in order to meet postnatal circulation demands, rodent cardiomyocytes transition from mononucleated forms to predominantly binucleated during the first week of life. However, the percentage of mononucleated cardiomyocytes in humans is ~ 65% throughout life [16], which differs from the transition in mice, indicating that the cytoskeletal architecture theory of mice cannot be directly applied to humans.

In summary, this is the first study to demonstrate a relationship between age and oxidative DNA damage in human cardiomyocytes. The results suggested that oxidative DNA damage in human cardiomyocytes is insufficient to fully explain the decreased proliferation activity in the postnatal period, which could be due to both the well-organized architecture of cardiomyocytes and their nuclear integrity. Heart failure therapy remains challenging and requires more intensive investigation, especially in human models.

## Supporting Information

S1 Fig Representative Echocardiography of patient with VSD. (JPG)

S1 Table Individual information of all included patients. (XLS)
