# Supplementary figures and images for: Age-Dependent Oxidative DNA Damage Does Not Correlate with Reduced Proliferation of Cardiomyocytes in Humans
Source: PLoS One. 2017 Jan 18;12(1):e0170351. doi: 10.1371/journal.pone.0170351 (PMC5242470; doi:10.1371/journal.pone.0170351)

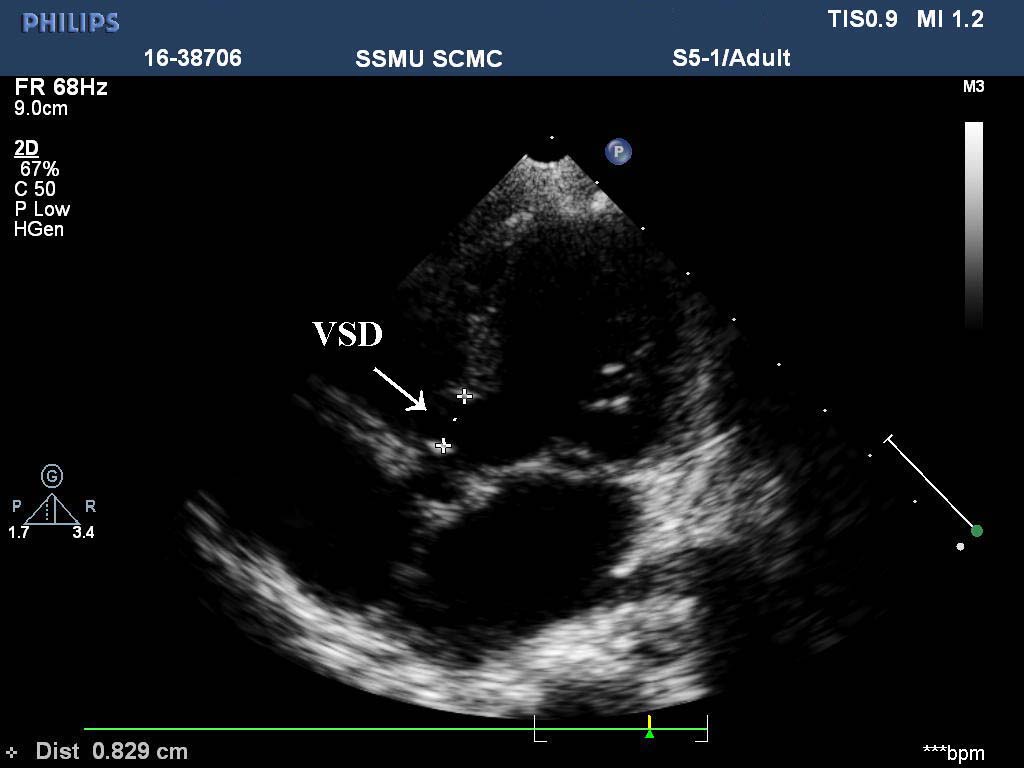

Supplement: S1 Fig — (JPG) [file pone.0170351.s001.jpg]
